# CNS Mitochondria‐Derived Vesicle in Blood: Potential Biomarkers for Brain Mitochondria Dysfunction

**DOI:** 10.1002/acn3.70060

**Published:** 2025-04-25

**Authors:** Qi Liu, Wentao Chen, Yahong Wu, Zhen Guo, Jun Chen, Chen Tian, Pan Wang, Shaopeng Zeng, Bin Xu, Jing Duan, Shilong Han, Xiao Xiong, Jing Zhang

**Affiliations:** ^1^ Department of Pathology The First Affiliated Hospital of Zhejiang University School of Medicine Hangzhou Zhejiang China; ^2^ The Central Laboratory The First Affiliated Hospital of Zhejiang University School of Medicine Hangzhou Zhejiang China; ^3^ National Human Brain Bank for Health and Disease The First Affiliated Hospital of Zhejiang University School of Medicine Hangzhou Zhejiang China

**Keywords:** Alzheimer's diseases, mitochondria‐derived vesicles, mitochondrial dysfunction, Parkinson's diseases

## Abstract

**Objective:**

Mitochondrial dysfunction is a hallmark of neurodegenerative diseases like Alzheimer's (AD) and Parkinson's (PD). Our goal was to develop practical, noninvasive methods to assess mitochondrial status through the detection of mitochondria‐derived vesicles (MDVs).

**Methods:**

We explored blood‐borne MDVs, a recently identified class of extracellular vesicles, as potential biomarkers for CNS mitochondrial status.

**Results:**

The study identified MDVs from neurons, astrocytes, and oligodendrocytes specifically in human plasma. A novel nanoflow cytometry was developed to evaluate the level of neuron‐, astrocyte‐, and oligodendrocyte‐derived MDVs in plasma in AD and PD patients. Importantly, analyses of discovery and validation cohorts revealed significantly lower brain cell‐specific MDVs in AD and PD patients compared to healthy controls.

**Interpretation:**

This study suggests that blood MDVs could serve as noninvasive biomarkers for mitochondrial dysfunction in AD, PD, and beyond, potentially aiding in monitoring mitochondrial‐focused therapies for neurological disorders.

**Abbreviations**
AD: Alzheimer's disease
AUC: area under curve
Aβ: amyloid‐beta
CNS: central nervous system
CSF: cerebrospinal fluid
CypD: cyclophilin D
EV: extracellular vesicle
GABA: gamma‐aminobutyric acid
MDD: major depressive disorder
MDV: mitochondria‐derived vesicle
MVB: multivesicular bodies
NTA: nanoparticle tracking analysis
OXPHOS: oxidative phosphorylation
PD: Parkinson's disease
ROC: receiver operating characteristic
ROS: reactive oxygen species
STED: stimulated emission depletion microscopy
STORM: stochastic optical reconstruction microscopy
TCA: tricarboxylic acid
TEM: transmission electron microscope
UC: ultracentrifugation

## 1 Introduction

Mitochondrial dysfunction is a hallmark of many human diseases, including neurodegenerative disorders like Alzheimer's Disease (AD) and Parkinson's disease (PD) [1]. Pharmacological approaches targeting mitochondrial components show promise in improving mitochondrial health by modulating dynamics, enhancing biogenesis, and reducing oxidative stress, with promising candidates including antioxidants and insulin sensitizers [2]. Additionally, dietary changes and advanced genetic techniques like CRISPR/Cas9 offer strategies to mitigate mitochondrial dysfunction, which is linked to aging, cancer, metabolic disorders, and neurodegeneration [3].

A newly proposed potential mitochondrial therapy involves mitochondrial transfer, where mitochondrial components are exchanged between cells to improve function and reduce oxidative stress [4]. Studies have suggested that mitochondrial transfer can prevent neuronal death in PD model rats lesioned with 6‐hydroxydopamine [5]. One method of mitochondrial transfer utilizes mitochondria‐derived vesicles (MDVs). MDVs are released by mitochondria and act as a defense mechanism by removing damaged proteins and preventing mitochondrial failure [6]. Besides mechanistic investigations, MDV, similar in size to exosomes, is a type of extracellular vesicle (EVs) also found in human blood and may serve as a biomarker for neurological diseases. For example, altered mitochondrial protein levels in neuronal EVs have been observed in major depressive disorder (MDD), potentially aiding in diagnosis and monitoring [7]. However, despite the fact that we as well as others have identified astrocytic and oligodendroglial EVs, in addition to neuronal EVs, in human plasma [8, 9], the alteration of CNS‐derived MDVs in AD and PD and whether MDVs can be detected in human blood remains elusive.

In this study, with a well‐established protocol, we identified neuron‐, astrocyte‐, and oligodendroglia‐derived MDVs in human plasma. Additionally, we developed a sensitive and rapid flow cytometry‐based technology to evaluate the levels of CNS cell‐specific MDVs in the peripheral plasma. This investigation demonstrated that all three types of EVs contain mitochondrial proteins, with substantial alterations observed in PD and AD patients.

## 2 Methods

### 2.1 Participants

Ethical approval for human blood collection was obtained from the Institutional Review Board at the First Affiliated Hospital of Zhejiang University School of Medicine, Hangzhou, Zhejiang, China (2022‐043). The discovery cohort included 41 PD patients, 46 ad patients, along with 62 age‐ and sex‐matched healthy controls (HC), enrolled from the First Affiliated Hospital of Zhejiang University School of Medicine (Table 1). The validation cohort comprised 51 PD patients, 66 ad patients, and 65 age‐ and sex‐matched HC, enrolled from the First Affiliated Hospital of Zhejiang University School of Medicine and Peking Union Medical College Hospital (Table 1). Written informed consent was obtained from all participants prior to blood collection. A comprehensive summary of the participants' clinical characteristics is provided in Table 1.

**TABLE 1:** Characteristics of discovery and validation cohort.

	Discovery cohort (*n* = 149)			Validation cohort (*n* = 182)		
	HC	PD	AD	HC	PD	AD
Number	62	41	46	65	51	66
Age						
Mean ± SD	72.16 ± 6.66	74.29 ± 9.86	69.68 ± 9.293	71.16 ± 7.87	69.83 ± 9.83	65.52 ± 8.19
Range	60–88	54–91	53–87	50–90	49–93	51–86
Sex (M:F)	29:33	22:19	20:26	29:36	28:23	32:34
MMSE						
Mean ± SD	28.68 ± 1.44^a^	—	20.54 ± 7.021^b^	28.72 ± 1.75^c^	—	18.80 ± 7.175^d^
Range	25–30	—	6–29	24–30	—	2–27

Abbreviations: AD, Alzheimer's disease; HC, healthy control; PD, Parkinson's disease.

^a^ *n* = 44.

^b^ *n* = 26.

^c^ *n* = 44.

^d^ *n* = 30.

### 2.2 Inclusion and Exclusion Criteria for AD Patients

All enrolled AD patients met the NINCDS‐ADRDA1 diagnostic criteria. The diagnosis was confirmed by two or more experienced neurologists independently. Inclusion criteria: (1) age between 45 and 90 years; (2) clinically diagnosed with dementia; (3) deficits in two or more cognitive domains; (4) gradual deterioration in memory and other cognitive functions; (5) no consciousness disturbances. Exclusion criteria: (1) visual field defects or ataxia; (2) focal neurological findings early in the disease course; (3) seizures or gait disturbances at the onset or early stage of the disease; (4) PET scan inconsistent with AD.

### 2.3 Inclusion and Exclusion Criteria for PD Patients

All enrolled PD patients met the MDS clinical diagnostic criteria for Parkinson's disease (MDS‐PD). The diagnosis was also confirmed by two or more experienced neurologists independently. Inclusion criteria: (1) age between 45 and 90 years; (2) clear and significant positive response to dopaminergic therapy; (3) resting tremor in the limbs; (4) presence of significant motor impairments. Exclusion criteria: (1) clear cerebellar abnormalities on examination; (2) clear cortical sensory loss; (3) normal functional neuroimaging of the presynaptic dopaminergic system.

### 2.4 Inclusion Criteria for Healthy Control (HC) Group

No known diagnosis of neurological or psychiatric diseases or disease history.

### 2.5 Blood Sample Collection

Following a standardized protocol, blood samples were collected in EDTA‐coated tubes (BD Vacutainer, cat. no. 367863). The samples underwent initial centrifugation at 1500 *g* for 15 min at 4°C to isolate plasma, followed by a secondary centrifugation step at 3200 *g* at 4°C for 15 min to remove residual cellular debris. Processed plasma samples were preserved at −80°C for further analysis.

### 2.6 Isolation of EVs From Plasma

To isolate EVs, 100 μL of plasma was subjected to sequential centrifugation. First, the sample was centrifuged at 12,000 *g* for 30 min at 4°C to remove cellular debris and larger particles. The supernatant was then collected, diluted with 1 mL of sterile‐filtered phosphate‐buffered saline (PBS), and ultracentrifuged at 100,000 *g* for 70 min to pellet EVs. The resulting pellet was washed by resuspending it in 1 mL of PBS and repeating the ultracentrifugation step (100,000 *g*, 70 min). Finally, purified EVs were resuspended in 100 μL of filtered PBS for downstream applications.

### 2.7 Western Blot

Western blot (WB) analysis was conducted to confirm the presence of mitochondrial‐associated proteins and EV‐specific markers. Following the previously described isolation protocol, 1 mL of plasma was processed to obtain EV fractions. The purified EVs were lysed on ice for 15 min using RIPA lysis buffer containing 1x PMSF. Subsequently, the EV lysates were mixed with protein loading buffer and denatured at 100°C for 10 min to prepare for electrophoretic separation. The proteins were separated using an 8%–16% Bis‐Tris Gel (M00660, Genescript) and subsequently transferred to a PVDF membrane (IPVH00010, Merck). The PVDF membrane was first treated with 5% skim milk for 1 h at room temperature to block nonspecific binding sites. Then, it was exposed to primary antibodies and maintained at 4°C overnight for immunoblotting. After washing with TBST, the PVDF membrane was incubated with IRDye 800CW Goat anti‐mouse or Goat anti‐rabbit secondary antibody (926‐32210, LI‐COR) for 1 h at room temperature. The primary antibodies used in this study were anti‐VDAC1 (ab14734, Abcam, 1:1000), anti‐NMDAR2A (MA5‐27693, Invitrogen, 1:1000), anti‐SLC1A2 (MAB20001, Abnova, 1:1000), anti‐CNPase (5664, CST, 1:1000), anti‐β‐actin (20536‐1‐AP, Proteintech, 1:1000), and anti‐Alix (2171, Cell Signaling Technology, 1:1000).

### 2.8 Nanoparticle Tracking Analysis

The quantification of particle numbers and size distribution of the EVs was conducted using nanoparticle tracking analysis (NTA; NS300, Nanosight). Three videos of 60 s each were recorded for each EV fraction, and all fractions underwent analysis using the same threshold setting. Analysis of the recorded videos was carried out utilizing NTA 3.1 software (Nanosight).

### 2.9 Transmission Electron Microscopy (TEM)

5 μL of EVs sample with a concentration of approximately 10^12^/mL was incubated on a carbon support film (XP‐CF300, copper grid, Shenzhen Guanpin) for 2 min. Excess liquid on the carbon film was blotted away using filter paper, followed by washing with 5 μL of PBS. Subsequently, 5 μL of 2% uranyl acetate was applied to stain the sample for 2 min. After removing excess liquid with filter paper, the sample was air‐dried at room temperature. Imaging was performed using a Talos L120C EM instrument (Thermo Fisher Scientific, FEI) operating at 120 kV, equipped with a CETA camera.

### 2.10 Cryogenic Electron Microscopy (Cryo‐EM)

(1) Sample preparation: Using a Vitrobot (Thermo Fisher) under 100% humidity at 4°C, 3 μL of EVs sample with a concentration of approximately 10^12^/mL was incubated on a glow‐discharged Quantifoil holey carbon grid (R2/1, 300 mesh, copper, Q85959). The sample was blotted with appropriate force and duration and rapidly frozen in liquid nitrogen‐cooled liquid ethane to form a vitrified amorphous ice layer, preserving the native structure of the sample. The grids were stored under liquid nitrogen conditions to prevent ice crystal formation and radiation damage. (2) Image acquisition: The EVs on the carbon grids were visualized using a 300 keV Titan Krios electron microscope (Thermo Fisher) equipped with a K2 Summit camera (Gatan). The Cryo‐EM images were acquired in counting mode using SerialEM at a nominal magnification of 33,000. Tilt‐series for cryo‐electron tomography were collected from −51° to 51° at 3° intervals, yielding 35 images per series. The pixel size was 3.6 Å, and the defocus range was set between −0.9 and − 1.5 μm.

### 2.11 Nanoflow Cytometry Analysis

The anti‐VDAC1/Porin antibody (ab14734, Abcam) was labeled with Zenon Alexa Fluor 647 mouse IgG Mouse2b labeling kit (Z25208, Invitrogen), the anti‐CNPase antibody (5664S, Cell Signaling Technology) was labeled with Zenon Alexa Fluor 488 rabbit labeling kit (Z25302, Invitrogen), the anti‐SLC1A2 antibody (MAB20001, Abnova) was labeled with Zenon Alexa Fluor 488 rabbit labeling kit (Z25302, Invitrogen), and the anti‐NMDAR2A antibody was labeled with Zenon Alexa Fluor 488 mouse IgG Mouse2b labeling kit (Z25202, Invitrogen), according to the manufacturer's protocol. Briefly, 5 μL EV samples were blocked with 2% BSA of the same volume at room temperature for 1 h, followed by the addition of 5 μL PBS (0.22 μm filtered). The fluorophore‐conjugated antibody (0.03 μg of each antibody per sample) was then added to the samples and incubated overnight at 4°C. The isotype IgG control of each antibody was labeled with the same procedure. The samples were fixed with 10 μL 4% PFA (0.22 μm filtered) at room temperature for 20 min before the nanoflow cytometry analysis with the CytoFLEX LX (Beckman). The stability of the assay was evaluated by testing the samples on days 1, 3, and 5. The samples were diluted linearly (1:1, 1:2, 1:4) to evaluate the accuracy of the assay.

### 2.12 Stochastic Optical Reconstruction Microscopy

The EV samples were labeled with conjugated antibodies as described above. After fixation using 4% PFA, the samples were immersed in 300 μL specialized Stochastic Optical Reconstruction Microscopy (STORM) imaging buffer (50 mM Tris–HCL, pH 8.0, 10 mM NaCl, and 10% Glucose). All images were acquired using the Nikon N‐STORM super‐resolution system (Nikon Instruments Inc.) equipped with a Nikon Eclipse Ti inverted microscope. The fluorophores Alexa 488 and Alexa 647 were excited by 488 and 647 nm semiconductor lasers, respectively.

### 2.13 Stimulated Emission Depletion Microscopy (STED)

10 μL of the EV samples enriched with ultracentrifugation were incubated with VDAC1 (ab14734, Abcam) or cytochrome c (ab13575, Abcam) at room temperature for 1 h. The samples were subsequently treated with the secondary antibody Abberior STAR RED (Abberior) at room temperature for 1 h before super‐resolution STED acquisition. Imaging procedures were conducted using the Abberior STEDYCON super‐resolution system controlled by Inspector acquisition software (Abberior, Göttingen, Germany). This nanoscopy platform was assembled on an Olympus IX83 inverted microscope framework, incorporating a UPlanSApo 100× oil immersion objective (numerical aperture 1.40; Olympus) and an Andor iXon Ultra 897 EMCCD detector for signal capture.

### 2.14 Statistical Analysis

All statistical analyses were performed with SPSS 23.0 (IBM, Chicago, IL) and Prism 9.0 (GraphPad Software, La Jolla, CA). Data comparisons were conducted using two‐tailed, unpaired Student's *t*‐tests for comparisons between two groups and one‐way ANOVA supplemented by Tukey's post hoc analysis for comparisons among multiple groups. The integrative analyses were conducted utilizing the binomial logistic regression method, with the level of neuron‐, astrocyte‐, and oligodendrocyte‐derived MDVs, as well as the ratio of cell‐specific MDVs to the total amount of EVs from each cell type. ROC curves were employed to evaluate the diagnostic performance, sensitivity, and specificity of differentiating PD or AD from HC. The optimal threshold for each ROC curve was determined as the point yielding the highest combined sensitivity and specificity.

## 3 Results

### 3.1 Identification of CNS Cell‐Specific Plasma MDVs

**FIGURE 1:**
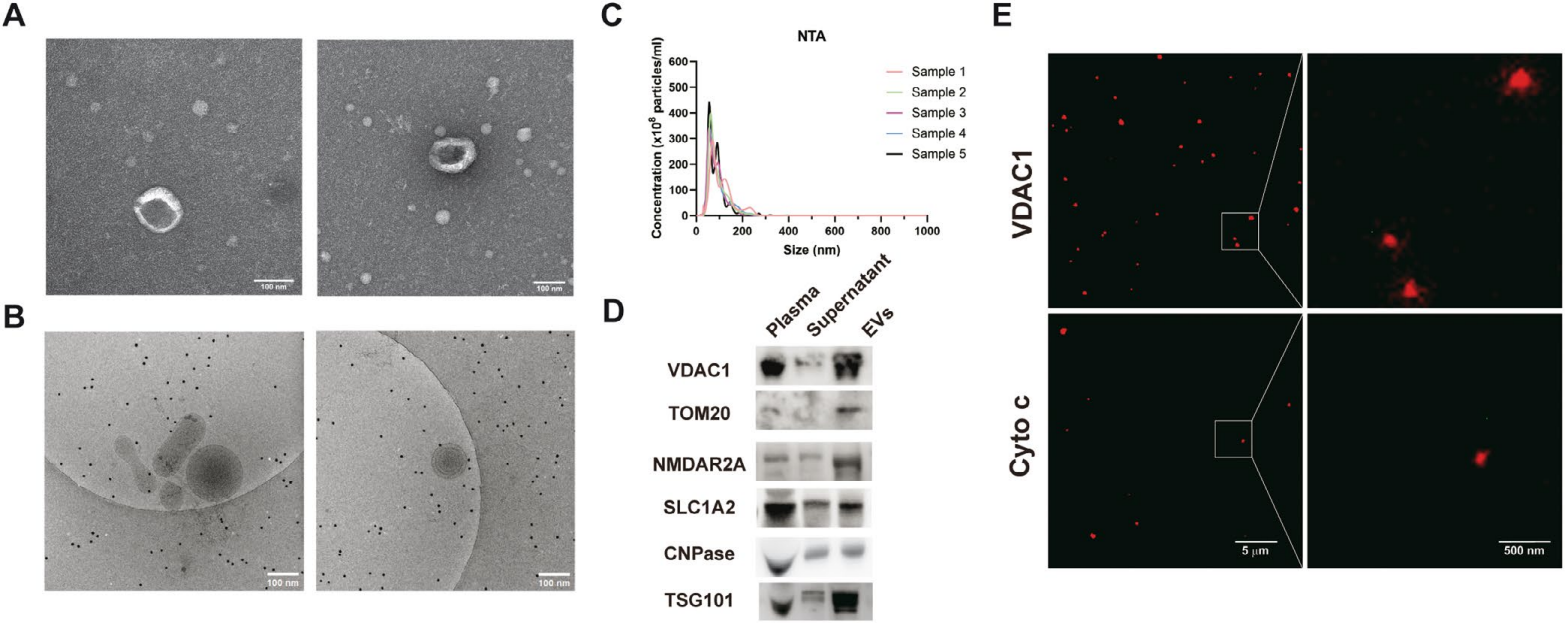
Characterization of EVs enriched by ultracentrifugation. (A) The structure of EVs revealed by TEM showed vesicles with a diameter of approximately 100 nm. (B) Cryo‐TEM revealed the double‐membrane structure of EVs. (C) NTA showed the population of EVs with a peak of approximately 70 nm (*n* = 5). (D) Mitochondria proteins and neuron, astrocyte, and oligodendrocyte marker proteins were present in the EVs fraction obtained from ultracentrifugation. (E) STED detects mitochondria proteins in the EVs obtained from plasma.

To ascertain the presence of CNS cell‐specific MDVs in human plasma, EVs were isolated following well‐established procedures from the samples of HC [8]. TEM and cryo‐EM analysis revealed that the EVs exhibited a characteristic diameter of approximately 100 nm, displaying typical exosome membrane structures (Figure 1A,B). NTA analysis further confirmed that the sizes of EVs predominantly ranged from 30 to 150 nm, aligning with the established size range for exosomes (Figure 1C). Western blot analysis demonstrated that the EVs isolated from plasma contained mitochondria‐related proteins, including TOM20 and VDAC1 (Figure 1D). The EVs isolated from ultracentrifugation were also enriched for NMDAR2A, SLC1A2, and CNPase, markers of neurons, astrocytes, and oligodendrocytes (Figure 1D), respectively. Furthermore, STED detected mitochondrial protein VDAC1 in the isolated EVs, suggesting that parts of EVs contain mitochondrial proteins or fragments of mitochondria, i.e., MDVs (Figure 1E).

**FIGURE 2:**
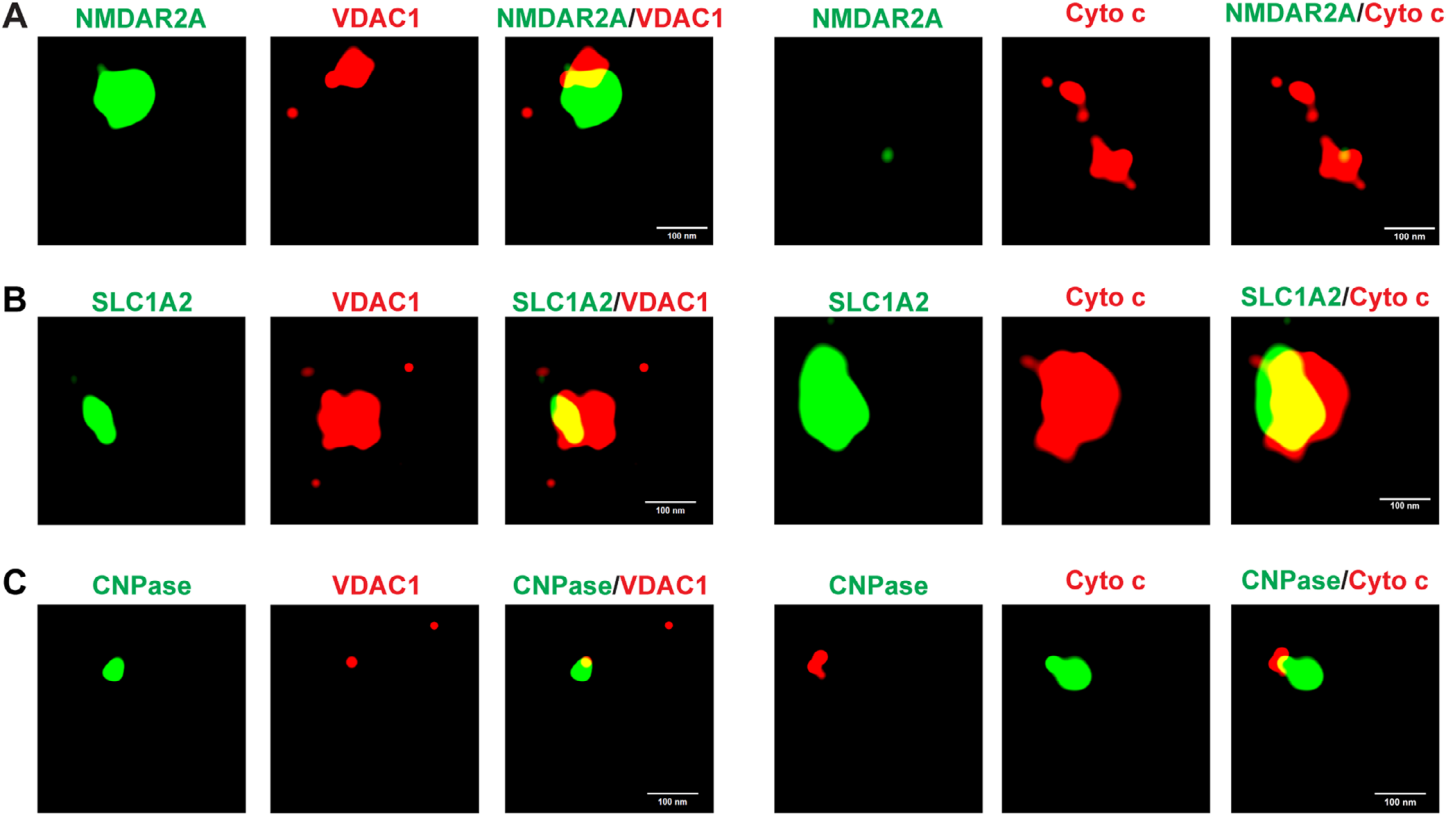
Neuron, astrocyte, and oligodendrocyte marker proteins are colocalized with mitochondrial proteins on EVs. (A) STORM imaging revealed the colocalization of neuron marker NMDAR2A (green) with mitochondria marker VDAC1 (red) or cytochrome c (red) on EVs. Scale bar = 100 nm. (B) STORM imaging confirmed the presence of mitochondria protein VDAC1 (red) or cytochrome c (red) on SLC1A2^+^ EVs. Scale bar = 100 nm. (C) STORM imaging confirmed the colocalization of oligodendrocyte marker CNPase (green) and mitochondrial protein VDAC1 (red) or cytochrome c (red). Scale bar = 100 nm.

STORM was utilized to further investigate the presence of CNS‐derived MDVs in human plasma from HCs. This analysis revealed that NMDAR2A, SLC1A2, and CNPase—markers expressed by CNS neurons, astrocytes, and oligodendrocytes, correspondingly—were readily colocalized with mitochondria proteins VDAC1 and cytochrome c, which further indicates the presence of neuron‐, astrocyte‐, and oligodendrocyte‐derived MDVs in plasma (Figure 2A–C). Furthermore, to confirm the co‐localization of mitochondria markers with neuron‐, astrocyte‐, and oligodendrocyte‐specific markers, the three‐dimensional videos were constructed and provided in Videos [Link], [Link], [Link], [Link], [Link], [Link].

### 3.2 Development of Assays for CNS‐Derived MDVs

**FIGURE 3:**
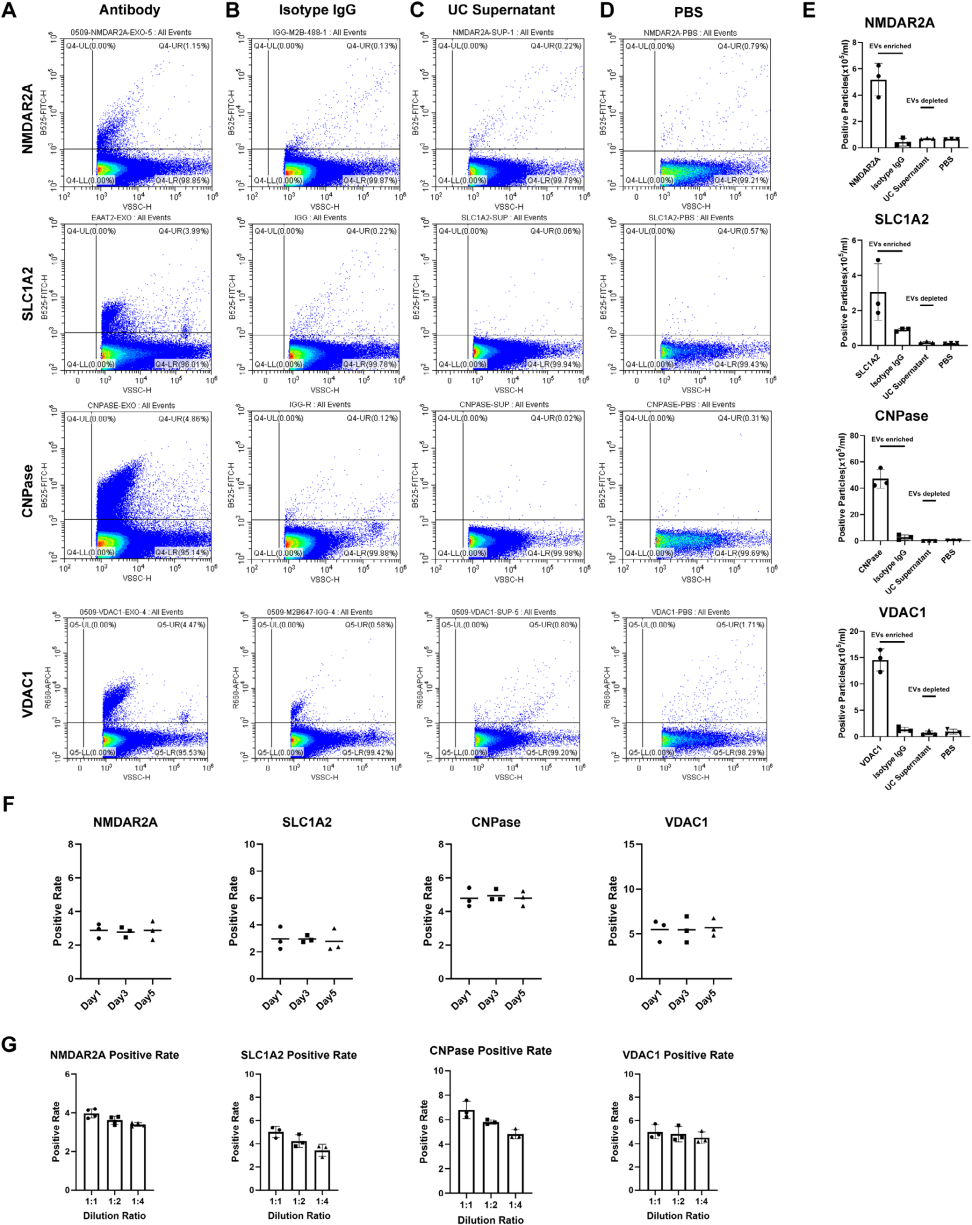
Development of novel flow cytometry‐based assays for CNS‐derived MDVs on plasma vesicles. (A) Examples of flow cytometry showing populations of EVs positive for each indicated marker. (B) Examples of plasma EVs samples labeled with fluorophore‐conjugated immunoglobulin G isotype control for the indicated marker antibody. (C) Examples of ultracentrifugation supernatant of plasma labeled with indicated marker conjugated with fluorophores. (D) Examples of PBS incubated with antibody conjugated with fluorophores. (E–G) Summary data from experiments demonstrating the specificity of EV assays (*n* = 3) (E), stability of labeling of EVs on 3 separate days (day 1, day 3, day 5) for indicated markers (F), and linearity in different dilutions of EVs samples (G).

To explore whether CNS‐derived MDVs could serve as biomarkers for monitoring mitochondrial dysfunction, we optimized a nanoflow cytometry‐based assay previously developed for CSF [10] and plasma [8]. This assay was then used to assess patients with AD and PD, two diseases well‐known for mitochondrial dysfunction as a key pathophysiological process. To facilitate the detection and analysis of MDVs, the VSSC model of CytoFLEX LX was utilized for the detection. As shown in Figure 3A, VDC1^+^ MDVs were detected in plasma, while negligible positive signals were detected in the isotype immunoglobulin G (IgG) control group, UC supernatant, and dilution buffer (Figure 3A–E). Antibodies targeting NMDAR2A, SLC1A2, and CNPase enabled the labeling of neuron‐, astrocyte‐, and oligodendrocyte‐derived EVs, respectively, in plasma [8, 9, 11]. As expected, minimal positive signals were detected in the isotype IgG control group, UC supernatant, and dilution buffer. The combination of NMDAR2A, SLC1A2, and CNPase with VDAC1 was then utilized for the detection and analysis of neuron‐, astrocyte‐, and oligodendrocyte‐derived MDVs. These results verified the assay's specificity for detecting CNS‐derived MDVs. Additionally, the stability of the assay was investigated by labeling EVs with antibodies targeting VDAC1, CNPase, SLC1A2, and NMDAR2A followed by storage for 1, 3, and 5 days before detection. The results demonstrated consistent labeling percentages of EVs across different time points (Figure 3F). The linearity of dilution clearly proved the accuracy of the assay (Figure 3G).

### 3.3 Neuron‐Derived MDVs in PD and AD Patients

**FIGURE 4:**
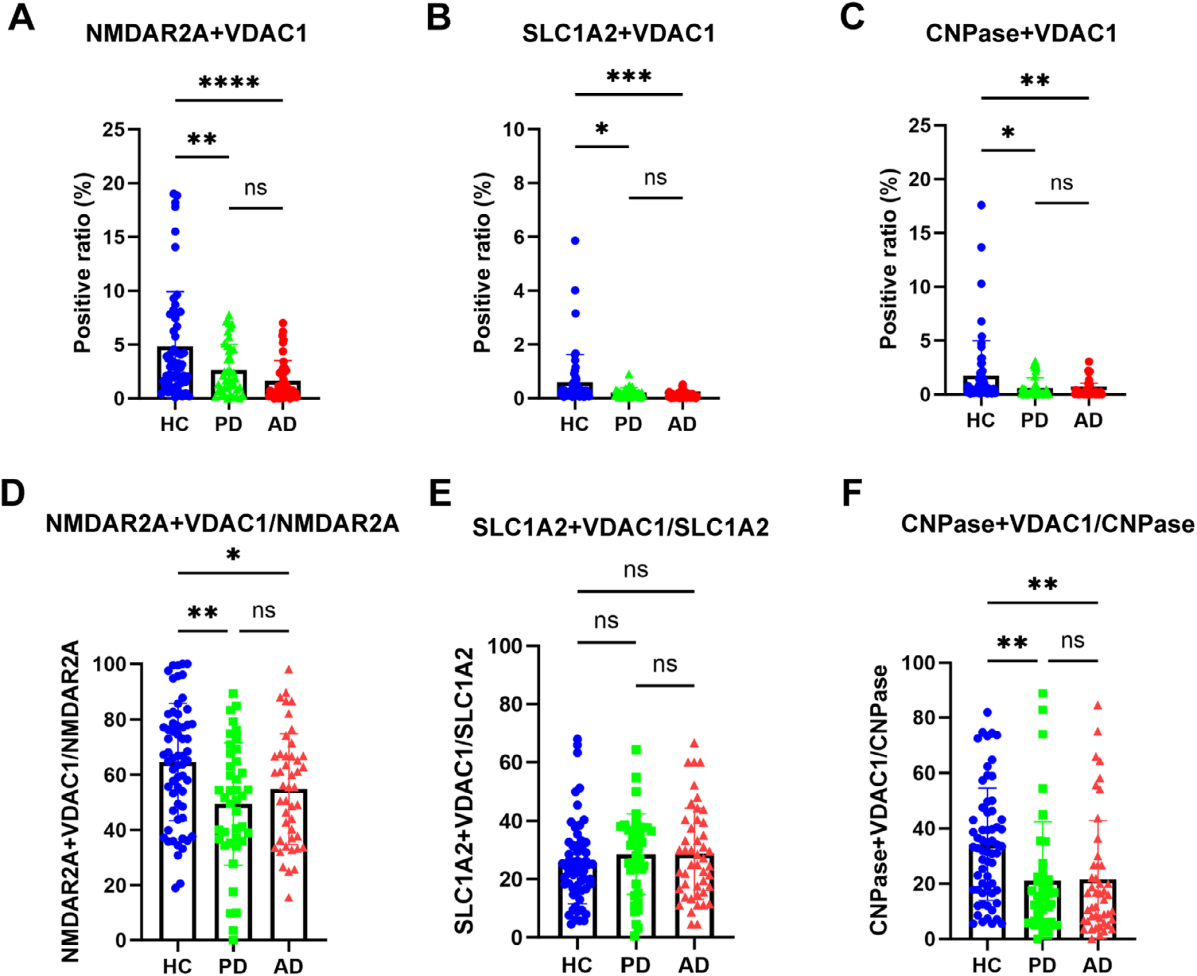
Performance of CNS‐derived MDVs in the discovery cohort. (A) The percentage of NMDAR2A^+^ and VDAC1^+^ EVs was lower in PD and AD patients than in healthy controls. (B) The level of SLC1A2^+^ and VDAC1^+^ EVs was lower in PD and AD patients than in healthy controls. (C) The level of CNPase^+^ and VDAC1^+^ EVs was lower in PD and AD patients than in healthy controls. (D) The percent of neuron‐derived MDVs in the total neuron‐derived EVs in the plasma of AD, PD patients, and HC in the discovery cohort. (E) The percent of astrocyte‐derived MDVs in the total astrocyte‐derived EVs in the plasma of AD, PD patients, and HC in the discovery cohort. (F) The percent of oligodendrocyte‐derived MDVs in the total oligodendrocyte‐derived EVs in the plasma of AD, PD patients, and HC in the discovery cohort. The *y*‐axis values in figure A–C represent the positive ratio of MDVs from neurons, astrocytes, or oligodendrocytes in the context of all EVs including CNS derived and non‐CNS derived. ns, not significant; *p < 0.05; **p < 0.01; ***p < 0.001; ****p < 0.0001. One‐way ANOVA followed by Tukey's multiple comparisons test.

**FIGURE 5:**
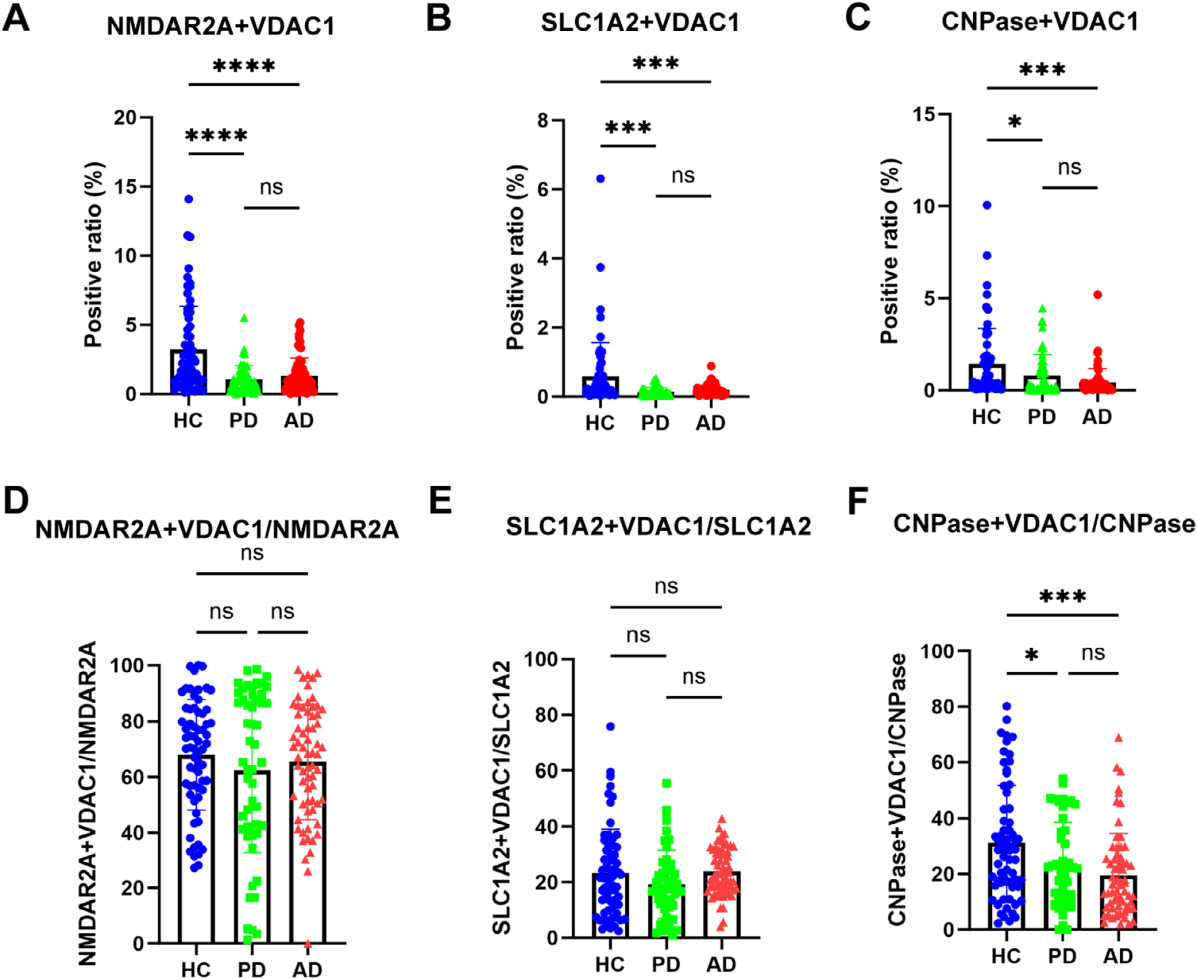
Performance of CNS‐derived MDVs in the validation cohort. (A) The level of NMDAR2A^+^ and VDAC1^+^ EVs was lower in PD and AD patients than in healthy controls. (B) The percentage of SLC1A2^+^ and VDAC1^+^ EVs was reduced in PD and AD patients compared to healthy controls. (C) The level of CNPase^+^ and VDAC1^+^ EVs was lower in PD and AD patients than in healthy controls. (D) The percent of neuron‐derived MDVs in the total neuron‐derived EVs in the plasma of AD, PD patients, and HC in the validation cohort. (E) The percent of astrocyte‐derived MDVs in the total astrocyte‐derived EVs in the plasma of AD, PD patients, and HC in the validation cohort. (F) The percent of oligodendrocyte‐derived MDVs in the total oligodendrocyte‐derived EVs in the plasma of AD, PD patients, and HC in the validation cohort. ns, not significant. *p < 0.05; ***p < 0.001; ****p < 0.0001. One‐way ANOVA followed by Tukey's multiple comparisons test.

To explore the possibility of neuron‐derived MDVs in monitoring mitochondrial dysfunction in PD and AD, discovery and validation samples collected from multiple medical centers were analyzed with the optimized nanoflow cytometry‐based assay. As shown in Figure 4A and Figure S1B, the levels of NMDAR2A^+^ and VDAC1^+^ EVs were significantly lower in PD and AD patients compared to HC in the discovery cohort. The results observed in the discovery cohort were largely replicated in the validation cohort (Figure 5A, Figure S1F). The ratio of neuronal MDVs to total neuronal EVs was also reduced in PD and AD patients compared to that of HC in the discovery cohort (Figure 4D, Figure S2A). These results indicate that the decrease in neuronal MDVs is not primarily due to changes in overall neuron‐derived EVs levels. However, while a similar trend was observed in the validation cohort, the statistical significance seen in the discovery cohort was not replicated (Figure 5D, Figure S2D). This discrepancy likely suggests that a reduction in total neuronal EVs may, at least in part, contribute to the observed changes in certain measured parameters.

To evaluate changes in neuron‐derived MDVs in PD and AD patients using an orthogonal approach, we conducted a statistical analysis of large‐field STORM images. Consistent with our results from nano‐flowcytometry, the analysis showed that the level of neuron‐derived MDVs was reduced in PD and AD patients compared to that of HC (Figure S3A,B).

### 3.4 Astrocyte‐Derived MDVs in PD and AD Patients

Like neuron‐derived MDVs, when astrocyte‐derived MDVs were evaluated in the discovery cohort, the levels of SLC1A2^+^ and VDAC1^+^ EVs were significantly decreased in both PD and AD patients (Figure 4B, Figure S1C). However, the ratio of astrocyte‐derived MDVs to the total astrocytic EVs was not altered in PD and AD patients compared to that of HC (Figure 4E, Figure S2B). Consistently, the results were also replicated in the validation cohort (Figure 5B,E, Figure S1G and Figure S2E).

Similar to neuronal MDVs, statistical analysis of STORM images with a large field also revealed that the levels of astrocyte‐derived MDVs were reduced in PD and AD patients compared to that of HC (Figure S3C,D).

### 3.5 Oligodendrocyte‐Derived MDVs in PD and AD Patients

To date, very little is known about mitochondrial dysfunction in oligodendrocytes, whether in PD or AD. However, as shown in Figure 4C and Figure S1D, the levels of oligodendrocyte‐derived MDVs were significantly lower in AD and PD patients compared to HCs. The ratio of oligodendrocyte‐derived MDVs to oligodendroglial EVs was also significantly reduced in the PD and AD patients compared to that of HC (Figure 4G, Figure S2C). These results indicate again that the decrease in oligodendrocyte‐derived MDVs is not primarily due to changes in overall EV levels. These results were validated as well in the validation cohort (Figure 5C,F, Figure S1H, Figure S2F).

Similar to neuronal and astrocyte‐derived MDVs, an independent assay—statistical analysis of large‐field STORM images—again demonstrated a reduced level of oligodendrocyte‐derived MDVs in PD and AD patients compared to that of HC (Figure S3E,F).

### 3.6 CNS‐Derived MDVs as Diagnostic Markers for PD and AD

**FIGURE 6:**
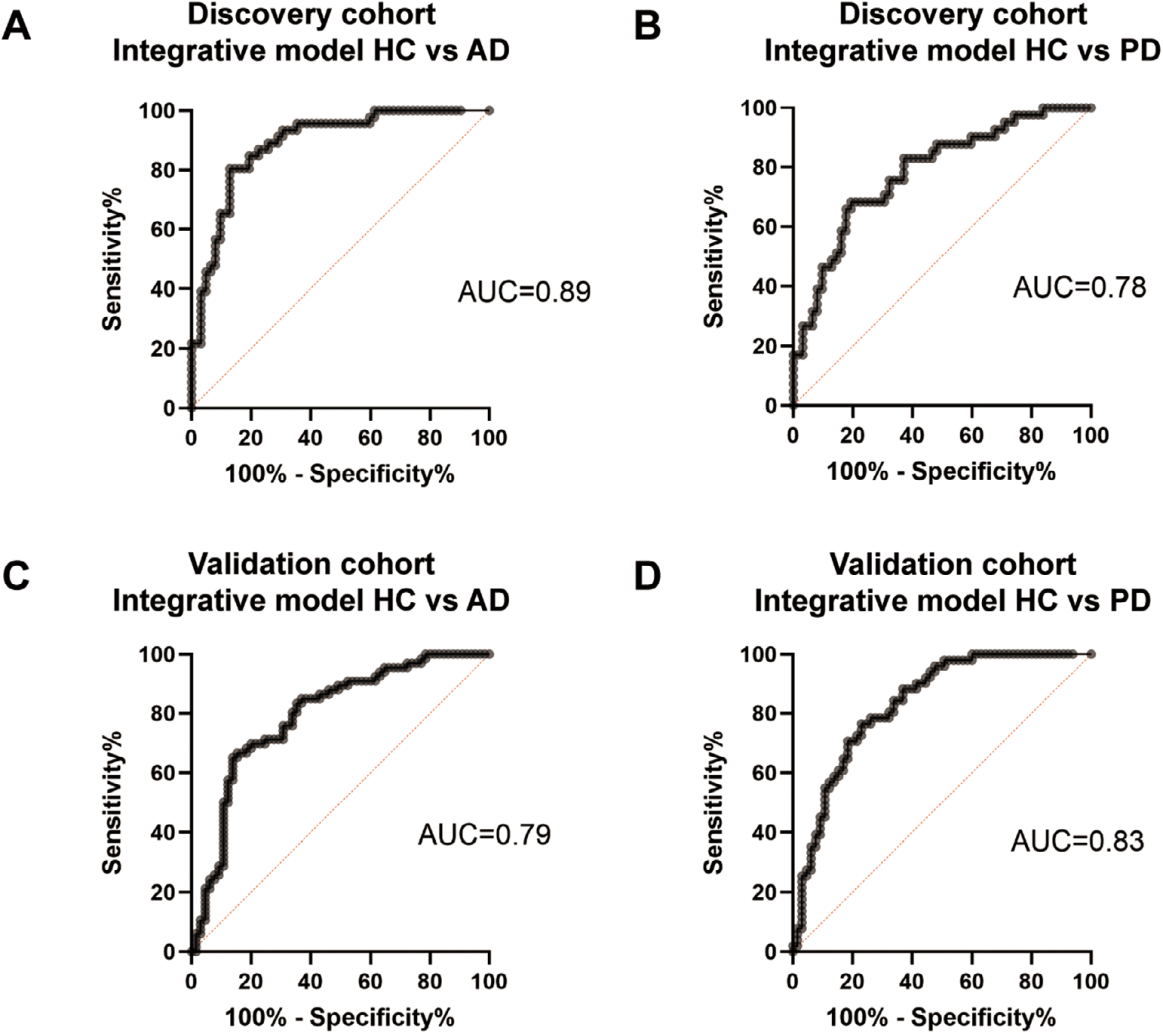
Performance of integrative model of CNS‐derived MDVs for diagnosis of AD and PD patients. (A) Performance of integrative model of CNS‐derived MDVs for distinguishing AD patients from HC in the discovery cohort. (B) Performance of integrative model of CNS‐derived MDVs for distinguishing PD patients from HC in the discovery cohort. (C) Performance of integrative model of CNS‐derived MDVs for distinguishing AD patients from HC in the validation cohort. (D) Performance of integrative model of CNS‐derived MDVs for distinguishing PD patients from HC in the validation cohort.

While individual MDV type showed limited area under the curve (AUC) values for the diagnosis of PD and AD (Table 2 and Table 3), combining them in a binomial logistic regression model significantly improved performance. This improvement was based on the level of neuron‐, astrocyte‐, and oligodendrocyte‐derived MDVs, as well as the ratio of cell‐specific MDVs to the total amount of EVs from each cell type. In the discovery cohort, the integrative model discriminated AD from HC with an AUC of 0.89 (95% CI = 0.83 to 0.95, sensitivity = 0.85, specificity = 0.81) (Figure 6A). Regarding PD and HC, the integrative model achieved an AUC of 0.78 (95% CI = 0.69–0.87), with a sensitivity of 0.76 and specificity of 0.68 (Figure 6B). In the validation cohort, the integrative model of CNS‐derived MDVs showed an AUC of 0.79 (95% CI = 0.72–0.87) for the discrimination of AD and HC, with a sensitivity of 0.71 and a specificity of 0.75 (Figure 6C). The AUC of the integrative model to discriminate PD and HC was 0.83 (95% CI = 0.76–0.91, sensitivity = 0.78, specificity = 0.74) in the validation cohort (Figure 6D).

**TABLE 2:** Performance of CNS‐derived MDV on the diagnosis of AD or PD.

	Discovery cohort		Validation cohort	
	HC versus AD	HC versus PD	HC versus AD	HC versus PD
ROC				
NMDAR2A^+^ VDAC1^+^	0.75 (0.66–0.85)	0.63 (0.52–0.74)	0.71 (0.63–0.80)	0.75 (0.66–0.84)
SLC1A2^+^ VDAC1^+^	0.84 (0.77–0.91)	0.62 (0.51–0.73)	0.63 (0.54–0.73)	0.74 (0.66–0.83)
CNPase^+^ VDAC1^+^	0.82 (0.73–0.90)	0.71 (0.60–0.82)	0.72 (0.64–0.81)	0.65 (0.55–0.75)

**TABLE 3:** Performance of CNS‐derived MDV on the differentiation of AD or PD from HC.

	Discovery cohort				Validation cohort			
	HC versus AD		HC versus PD		HC versus AD		HC versus PD	
	Sensitivity	Specificity	Sensitivity	Specificity	Sensitivity	Specificity	Sensitivity	Specificity
NMDAR2A^+^ VDAC1^+^	0.72	0.57	0.66	0.55	0.71	0.60	0.76	0.60
SLC1A2^+^ VDAC1^+^	0.89	0.60	0.63	0.52	0.73	0.51	0.82	0.51
CNPase^+^ VDAC1^+^	0.83	0.56	0.73	0.44	0.76	0.51	0.63	0.42

It should be noted that there was no significant correlation between age or gender and the levels of CNS‐derived MDVs in the discovery cohort or the validation cohort (Figure S4A–L). However, as shown in Figure S4M–R, the levels of MDVs derived from oligodendrocytes, astrocytes, and neurons were correlated with MMSE scores in AD patients and HC in the validation cohort but not in the discovery cohort.

## 4 Discussion

In this study, we identified neuron‐, astrocyte‐, and oligodendrocyte‐derived MDVs in the blood and successfully developed assays to evaluate these CNS‐specific MDVs. Additionally, we demonstrated that the levels of CNS‐derived MDVs in plasma are substantially decreased in PD and AD patients.

Neuron‐derived MDVs have been shown to be altered in the blood of patients with MDD [7]. However, the current study is the first to demonstrate the presence of astrocytic and oligodendroglial MDVs in human plasma. The MDVs identified are similar in size to exosomes, reinforcing that exosomes, derived from multivesicular bodies (MVB) [12], cannot be defined by size alone. Recent investigations suggest defining various vesicles based on the enrichment of specific proteins associated with EVs in addition to size [13].

MDVs usually carry TOM20 and VDAC1, two essential import channel proteins of the mitochondrial outer membrane, on the surface [14]. Additionally, specific oxidative phosphorylation (OXPHOS) complexes in the mitochondrial inner membrane, enzymes in the mitochondrial matrix such as PDH, and those of the tricarboxylic acid (TCA) cycle are also usually incorporated, making the MDVs a distinct type of EVs [14]. Despite the fact that MDVs have been recognized recently [15], their functions appear to be multifaceted. MDVs play an important role in diverse physiological processes, including mitochondria quality control, inter‐organelle cargo shuttle, and immune signaling [6]. It also appears that the generation of MDVs acts as an early response to mitochondria stress, preceding mitophagy to discharge damaged proteins and to eliminate oxidative stress [16]. The mutation of VPS35, an autosomal‐dominant gene associated with PD, increases its interaction with DLP1, which enhances the trafficking of MDVs to lysosomes for degeneration [17]. The dysregulation of this process may be involved in the pathogenesis of PD. MDVs released by stressed adipocytes, which contain oxidatively damaged particles, can enter circulation and be taken up by cardiomyocytes, where they trigger substantial ROS production [18].

A major observation of the investigation is that neuron‐, astrocyte‐, and oligodendrocyte‐derived MDVs are all reduced in both AD and PD patients compared to HCs (Figure 4). This finding aligns with multiple studies showing mitochondrial damage associated with PD and AD pathogenesis. For instance, in AD, the accumulation of amyloid‐beta (Aβ) can lead to the reduction of mitochondrial mass, impairment of mitochondrial biogenesis, and an imbalance of fission and fusion in neurons [19]. The interaction between cyclophilin D (CypD) and mitochondrial Aβ increases the permeability of the mitochondria membrane and invokes mitochondrial stress along with neuron death [20]. Similarly, the risk genes of PD, including SNCA, LRRK2, PINK1, and Parkin, are closely related to mitochondria quality [21]. Defects in mitochondria complex I activity and abnormalities in mtDNA have been consistently detected in PD patients [22]. The reduced expression of mtDNA‐encoded cytochrome c subunit I and decreased cytochrome c activity further support the role of mitochondrial dysfunction in PD [23].

Because most investigations into mitochondrial dysfunction in the context of AD and PD are conducted in neurons, two critical issues need to be discussed further. First, our results showed that the levels of astrocyte‐ and oligodendroglia‐derived MDVs were altered in AD and PD patients, indicating the simultaneous presence of abnormal mitochondrial function in astrocytes and oligodendrocytes in addition to neurons. Astrocytes play a pivotal role in maintaining metabolic homeostasis, energy supply, and neurotransmitter recycling [24]. An early study detected reduced TCA cycle activity in astrocytes in an AD mouse model, which leads to the deficiency of gamma‐aminobutyric acid (GABA) synthesis in the neurons and impaired transmitter recycling [25]. This dysfunction also enhances amyloid generation, further inducing oxidative stress and mitochondrial impairment in astrocytes [26, 27]. However, demonstration of astrocytic mitochondrial dysfunction directly in human brain tissue is needed in future investigations. In PD, Chen et al. reported a downregulation of mitochondrial OXPHOS protein expression in astrocytes from PD patients [28]. This study indicates that, like neurons, astrocytes in PD also exhibit mitochondrial impairment. Moreover, α‐synuclein, when internalized by astrocytes, can localize on the mitochondria and cause OXPHOS dysfunction, abnormal morphology, and disturbance of fission and fusion [29].

As with astrocytes, oligodendrocytes are critical in PD and AD pathogenesis. Recent studies have revealed that demyelination is an early event of AD, preceding amyloid and tau pathology [30]. Additionally, research using MRI has indicated abnormalities in white matter in PD. However, there has been limited research focusing on the mitochondrial function of oligodendrocytes in PD and AD. On the other hand, mitochondria play a crucial role in the differentiation, myelin formation, and hemostasis of oligodendrocytes [31]. Although further research is needed to confirm the oligodendroglial mitochondrial dysfunction in PD and AD, collectively, our results emphasize the crucial role of astrocytic and oligodendroglial mitochondrial function in neurodegenerative diseases. Given the critical roles of astrocytes and oligodendrocytes, targeting their mitochondria may provide novel therapeutic strategies for PD and AD in future investigations.

The second major point to discuss is that our investigation measured the number of MDVs, not necessarily the levels of mitochondrial proteins or mitochondrial function. Therefore, while the observed decrease in neuron‐, astrocyte‐, and oligodendrocyte‐derived MDVs may suggest mitochondrial dysfunction in PD and AD patients, more intensive studies are needed to examine mitochondrial functions in MDVs and human brain tissue, particularly in astrocytes and oligodendrocytes. Additionally, the mechanisms underlying the decreased MDVs in the blood require further investigation. Are these changes due to synthesis, trafficking, secretion of MDVs, or alterations in the blood–brain barrier that regulates the passage of MDVs? Regardless of the mechanisms involved, our study opens a potential avenue for evaluating cell‐specific mitochondrial dysfunction in PD, AD, and beyond. More specifically, these blood‐based but CNS cell‐specific mitochondrial biomarkers can be a robust tool for developing therapeutic strategies targeting CNS diseases.

Our study has several limitations, particularly the relatively small sample size. Although MDV alterations were not influenced by age or gender, our findings showed that the levels of neuron‐, astrocyte‐, and oligodendrocyte‐derived MDVs correlated with MMSE in the validation cohort but not in the discovery cohort. Further studies with larger cohorts, especially those with longitudinal follow‐up, are needed to better examine the relationship between MMSE and CNS‐derived MDVs.

In conclusion, our study not only identified neuron‐, astrocyte‐, and oligodendrocyte‐derived MDVs in human blood but also demonstrated that these cell‐specific MDVs were substantially decreased in PD and AD patients. While further research is needed to fully understand the mechanisms involved, these cell‐specific MDVs might serve as biomarkers for CNS mitochondrial function, especially when targeting mitochondrial therapies with supplements, medicine, or genetic manipulations.

## Author Contributions

J.Z., Q.L., X.X., Z.G., and C.T. conceptualized this study. Q.L., W.C., J.C., Z.G., S.Z., and S.H. contributed to the data curation. Q.L., W.C., P.W., and J.D. analyzed the data. Q.L., J.Z., X.X., and B.X. wrote the manuscript. All authors read and approved the final manuscript.

## Ethics Statement

Ethical approval for human blood collection in this study was granted by the Institutional Review Board at the First Affiliated Hospital of Zhejiang University School of Medicine (2022‐043) before participant enrollment. Written informed consent was obtained from all individuals before blood sample collection.

## Consent

The authors have nothing to report.

## Conflicts of Interest

The authors declare no conflicts of interest.

## Supporting information


**Figure S1.** The total number of MDVs and MDVs derived from neuron, astrocyte, and oligodendrocyte per milliliter of plasma (A) Total number of MDVs per milliliter plasma of AD, PD patients, and HC in the discovery cohort. (B) Total number of neuron‐derived MDVs per milliliter plasma of AD, PD patients, and HC in the discovery cohort. (C) Total number of astrocyte‐derived MDVs per milliliter plasma of AD, PD patients, and HC in the discovery cohort. (D) Total number of oligodendrocyte‐derived MDVs per milliliter plasma of AD, PD patients, and HC in the discovery cohort. (E) Total number of MDVs per milliliter plasma of AD, PD patients, and HC in the validation cohort. (F) Total number of neuron‐derived MDVs per milliliter plasma of AD, PD patients, and HC in the validation cohort. (G) Total number of astrocyte‐derived MDVs per milliliter plasma of AD, PD patients, and HC in the validation cohort. (H) Total number of oligodendrocyte‐derived MDVs per milliliter plasma of AD, PD patients, and HC in the validation cohort. ns, not significant; *p < 0.05; **p < 0.01; ***p < 0.001; ****p < 0.0001. One‐way ANOVA followed by Tukey’s multiple comparisons test.

**Figure S2.** Level of neuron‐, astrocyte‐, and oligodendrocyte‐derived EVs in AD, PD patients, and HC. (A) Level of neuron‐derived EVs in plasma of AD, PD patients, and HC in the discovery cohort. (B) Level of astrocyte‐derived EVs in plasma of AD, PD patients, and HC in the discovery cohort. (C) Level of oligodendrocyte‐derived EVs in plasma of AD, PD patients, and HC in the discovery cohort. (D) Level of neuron‐derived EVs in plasma of AD, PD patients, and HC in the validation cohort. (E) Level of astrocyte‐derived EVs in plasma of AD, PD patients, and HC in the validation cohort. (F) Level of oligodendrocyte‐derived EVs in plasma of AD, PD patients, and HC in the validation cohort. ns, not significant; *p < 0.05; **p < 0.01; ***p < 0.001; ****p < 0.0001. One‐way ANOVA followed by Tukey’s multiple comparisons test.

**Figure S3.** Statistical analysis of STORM image with large field. (A) Representative image of STORM image of neuron‐derived MDVs in the plasma of AD, PD patients, and HC. (B) Statistical analysis of STORM images of neuron‐derived MDVs in the plasma of AD, PD patients, and HC. (C) Representative image of STORM image of astrocyte‐derived MDVs in the plasma of AD, PD patients, and HC. (D) Statistical analysis of STORM images of astrocyte‐derived MDVs in the plasma of AD, PD patients, and HC. (E) Representative image of STORM image of oligodendrocyte‐derived MDVs in the plasma of AD, PD patients, and HC. (F) Statistical analysis of STORM images of oligodendrocyte‐derived MDVs in the plasma of AD, PD patients, and HC. ns, not significant. * *p* < 0.05; **p < 0.01; ***p < 0.001; ****p < 0.0001. One‐way ANOVA followed by Tukey’s multiple comparisons test.

**Figure S4.** Correlation between the level of CNS‐derived MDVs and age, gender, and MMSE. (A) Correlation between age and neuron‐derived MDVs in the discovery cohort. (B) Correlation between age and astrocyte‐derived MDVs in the discovery cohort. (C) Correlation between age and oligodendrocyte‐derived MDVs in the discovery cohort. (D) Correlation between age and neuron‐derived MDVs in the validation cohort. (E) Correlation between age and astrocyte‐derived MDVs in the validation cohort. (F) Correlation between age and oligodendrocyte‐derived MDVs in the validation cohort. (G) Neuron‐derived MDVs in female and male subjects of AD, PD patients, and HC in the discovery cohort. (H) Astrocyte‐derived MDVs in female and male subjects of AD, PD patients, and HC in the discovery cohort. (I) Oligodendrocyte‐derived MDVs in female and male subjects of AD, PD patients, and HC in the discovery cohort. (J) Neuron‐derived MDVs in female and male subjects of AD, PD patients, and HC in the validation cohort. (K) Astrocyte‐derived MDVs in female and male subjects of AD, PD patients, and HC in the validation cohort. (L) Oligodendrocyte‐derived MDVs in female and male subjects of AD, PD patients, and HC in the validation cohort. (M) Correlation between MMSE and neuron‐derived MDVs in the discovery cohort. (N) Correlation between MMSE and astrocyte‐derived MDVs in the discovery cohort. (O) Correlation between MMSE and oligodendrocyte‐derived MDVs in the discovery cohort. (P) Correlation between MMSE and neuron‐derived MDVs in the validation cohort. (Q) Correlation between MMSE and astrocyte‐derived MDVs in the validation cohort. (R) Correlation between MMSE and oligodendrocyte‐derived MDVs in the validation cohort.

**Video S1.** 3D STORM showed colocalization of NMDAR2A and VDAC1 in the extracellular vesicle.

**Video S2.** 3D STORM showed colocalization of NMDAR2A and Cytochrome C in the extracellular vesicle.

**Video S3.** 3D STORM showed colocalization of SLC1A1 and VDAC1 in the extracellular vesicle.

**Video S4.** 3D STORM showed colocalization of SLC1A1 and Cytochrome C in the extracellular vesicle.

**Video S5.** 3D STORM showed colocalization of CNPase and VDAC1 in the extracellular vesicle.

**Video S6.** 3D STORM showed colocalization of CNPase and Cytochrome C in the extracellular vesicle.

## Data Availability

The datasets used or analyzed during the current study are available from the corresponding author on reasonable request.
