# Supplementary figures and images for: CNS Mitochondria‐Derived Vesicle in Blood: Potential Biomarkers for Brain Mitochondria Dysfunction
Source: Ann Clin Transl Neurol. 2025 Apr 25;12(7):1312–23. doi: 10.1002/acn3.70060 (PMC12257142; doi:10.1002/acn3.70060)

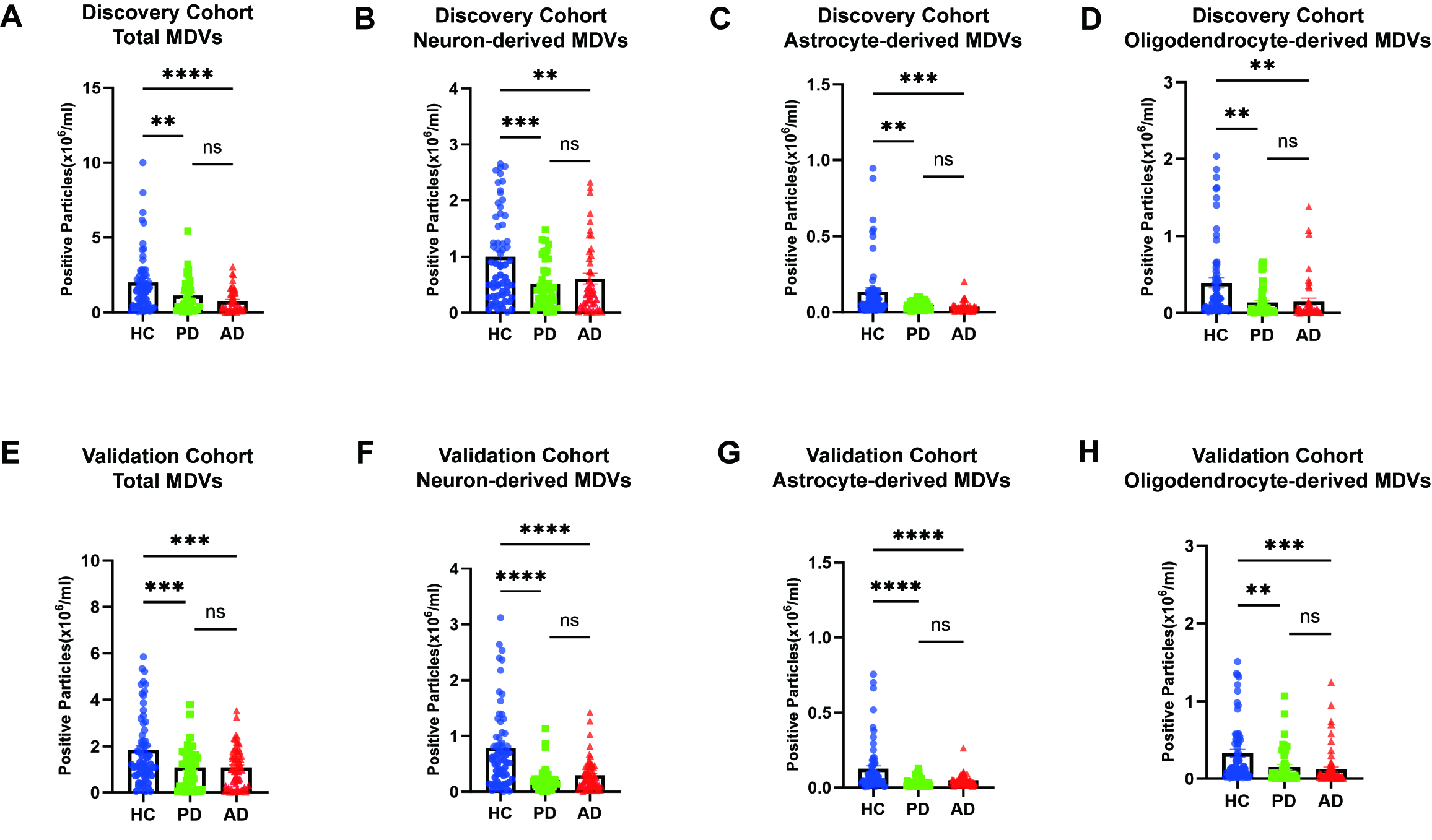

Supplement: Supplementary file 1 — Figure S1. The total number of MDVs and MDVs derived from neuron, astrocyte, and oligodendrocyte per milliliter of plasma (A) Total number of MDVs per milliliter plasma of AD, PD patients, and HC in the discovery cohort. (B) Total number of neuron‐derived MDVs per milliliter plasma of AD, PD patients, and HC in the discovery cohort. (C) Total number of astrocyte‐derived MDVs per milliliter plasma of AD, PD patients, and HC in the discovery cohort. (D) Total number of oligodendrocyte‐derived MDVs per milliliter plasma of AD, PD patients, and HC in the discovery cohort. (E) Total number of MDVs per milliliter plasma of AD, PD patients, and HC in the validation cohort. (F) Total number of neuron‐derived MDVs per milliliter plasma of AD, PD patients, and HC in the validation cohort. (G) Total number of astrocyte‐derived MDVs per milliliter plasma of AD, PD patients, and HC in the validation cohort. (H) Total number of oligodendrocyte‐derived MDVs per milliliter plasma of AD, PD patients, and HC in the validation cohort. ns, not significant; *p < 0.05; **p < 0.01; ***p < 0.001; ****p < 0.0001. One‐way ANOVA followed by Tukey’s multiple comparisons test. [file ACN3-12-1312-s005.tif]

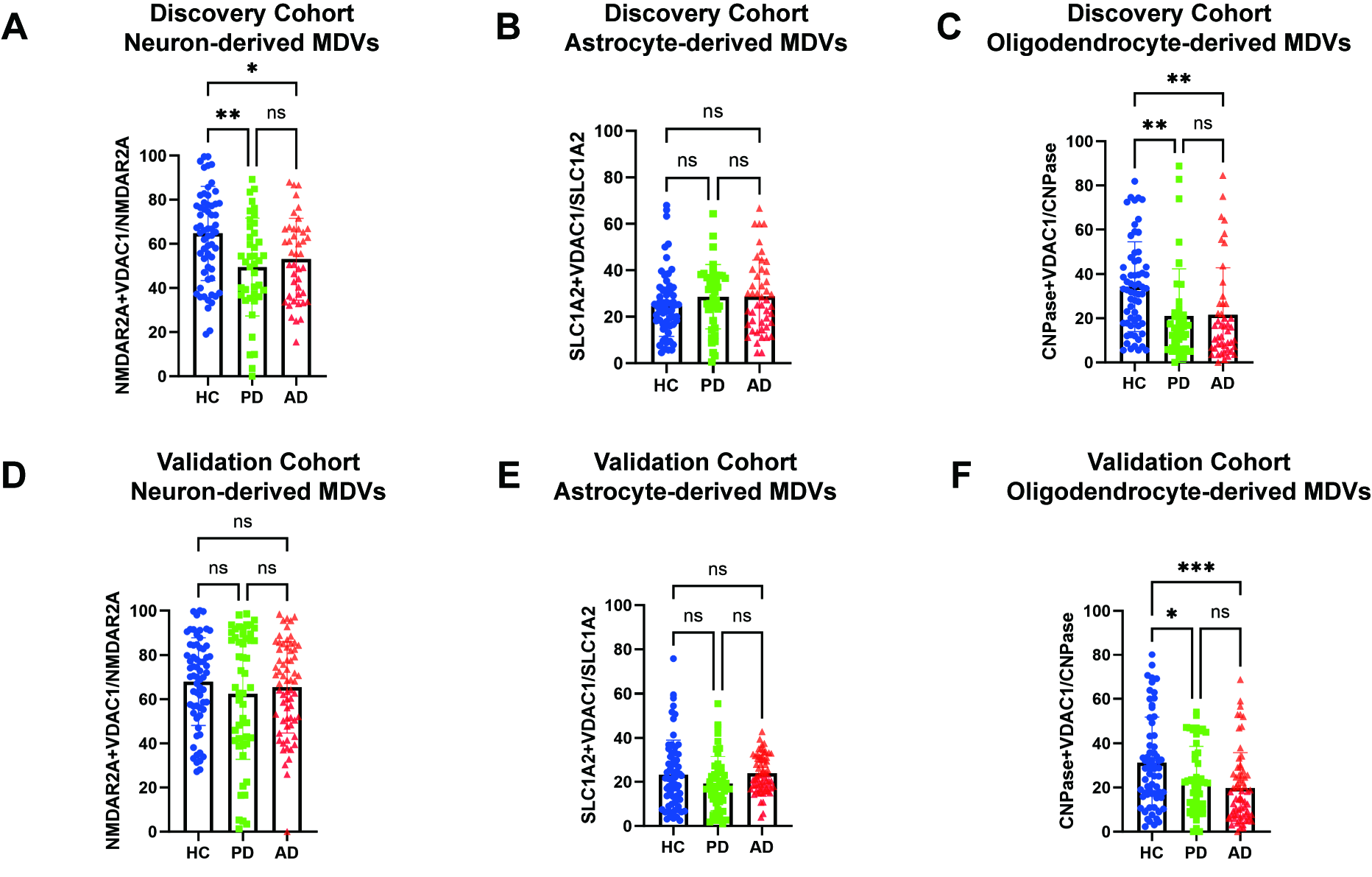

Supplement: Supplementary file 2 — Figure S2. Level of neuron‐, astrocyte‐, and oligodendrocyte‐derived EVs in AD, PD patients, and HC. (A) Level of neuron‐derived EVs in plasma of AD, PD patients, and HC in the discovery cohort. (B) Level of astrocyte‐derived EVs in plasma of AD, PD patients, and HC in the discovery cohort. (C) Level of oligodendrocyte‐derived EVs in plasma of AD, PD patients, and HC in the discovery cohort. (D) Level of neuron‐derived EVs in plasma of AD, PD patients, and HC in the validation cohort. (E) Level of astrocyte‐derived EVs in plasma of AD, PD patients, and HC in the validation cohort. (F) Level of oligodendrocyte‐derived EVs in plasma of AD, PD patients, and HC in the validation cohort. ns, not significant; *p < 0.05; **p < 0.01; ***p < 0.001; ****p < 0.0001. One‐way ANOVA followed by Tukey’s multiple comparisons test. [file ACN3-12-1312-s010.tif]

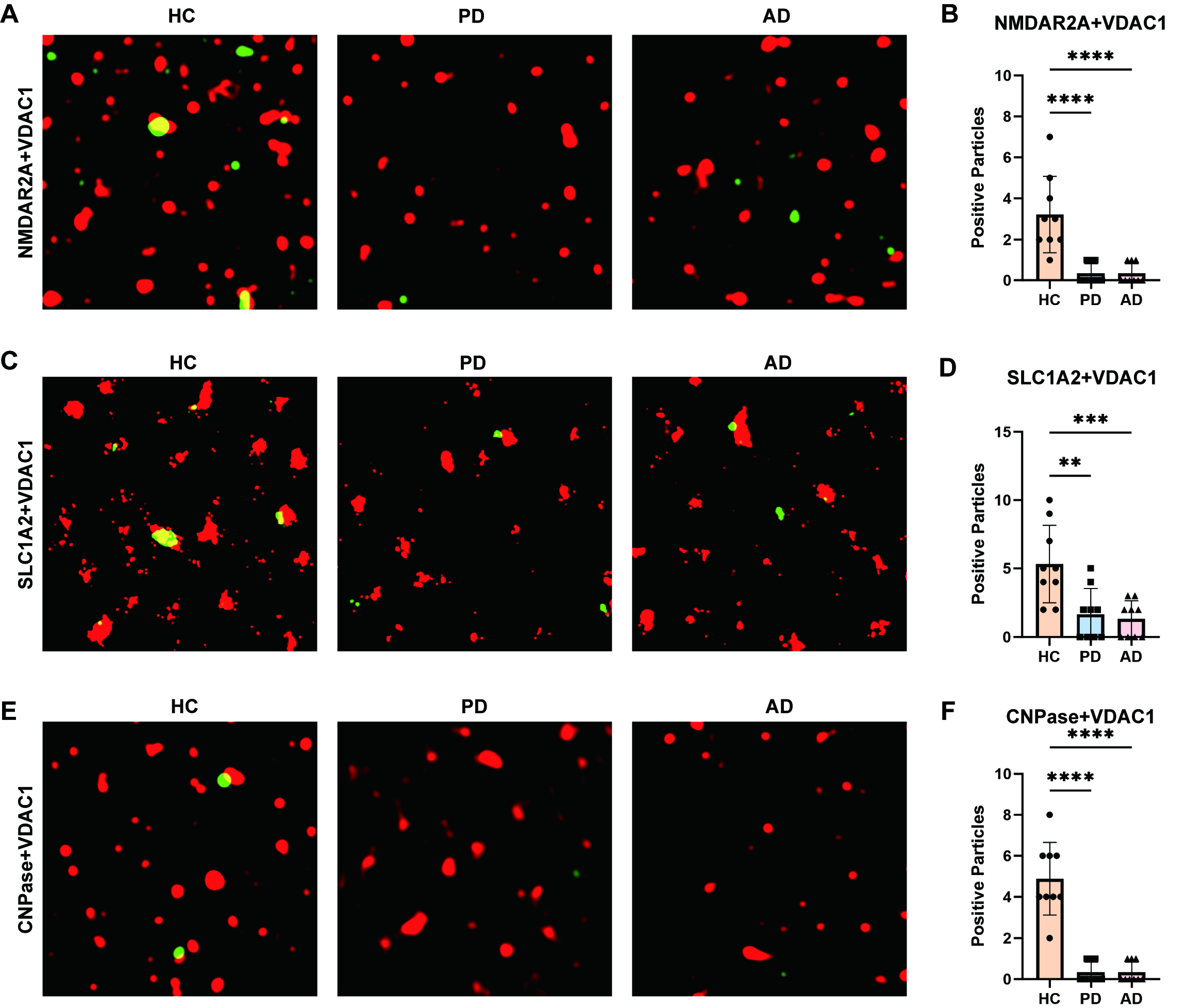

Supplement: Supplementary file 3 — Figure S3. Statistical analysis of STORM image with large field. (A) Representative image of STORM image of neuron‐derived MDVs in the plasma of AD, PD patients, and HC. (B) Statistical analysis of STORM images of neuron‐derived MDVs in the plasma of AD, PD patients, and HC. (C) Representative image of STORM image of astrocyte‐derived MDVs in the plasma of AD, PD patients, and HC. (D) Statistical analysis of STORM images of astrocyte‐derived MDVs in the plasma of AD, PD patients, and HC. (E) Representative image of STORM image of oligodendrocyte‐derived MDVs in the plasma of AD, PD patients, and HC. (F) Statistical analysis of STORM images of oligodendrocyte‐derived MDVs in the plasma of AD, PD patients, and HC. ns, not significant. * p < 0.05; **p < 0.01; ***p < 0.001; ****p < 0.0001. One‐way ANOVA followed by Tukey’s multiple comparisons test. [file ACN3-12-1312-s001.tif]

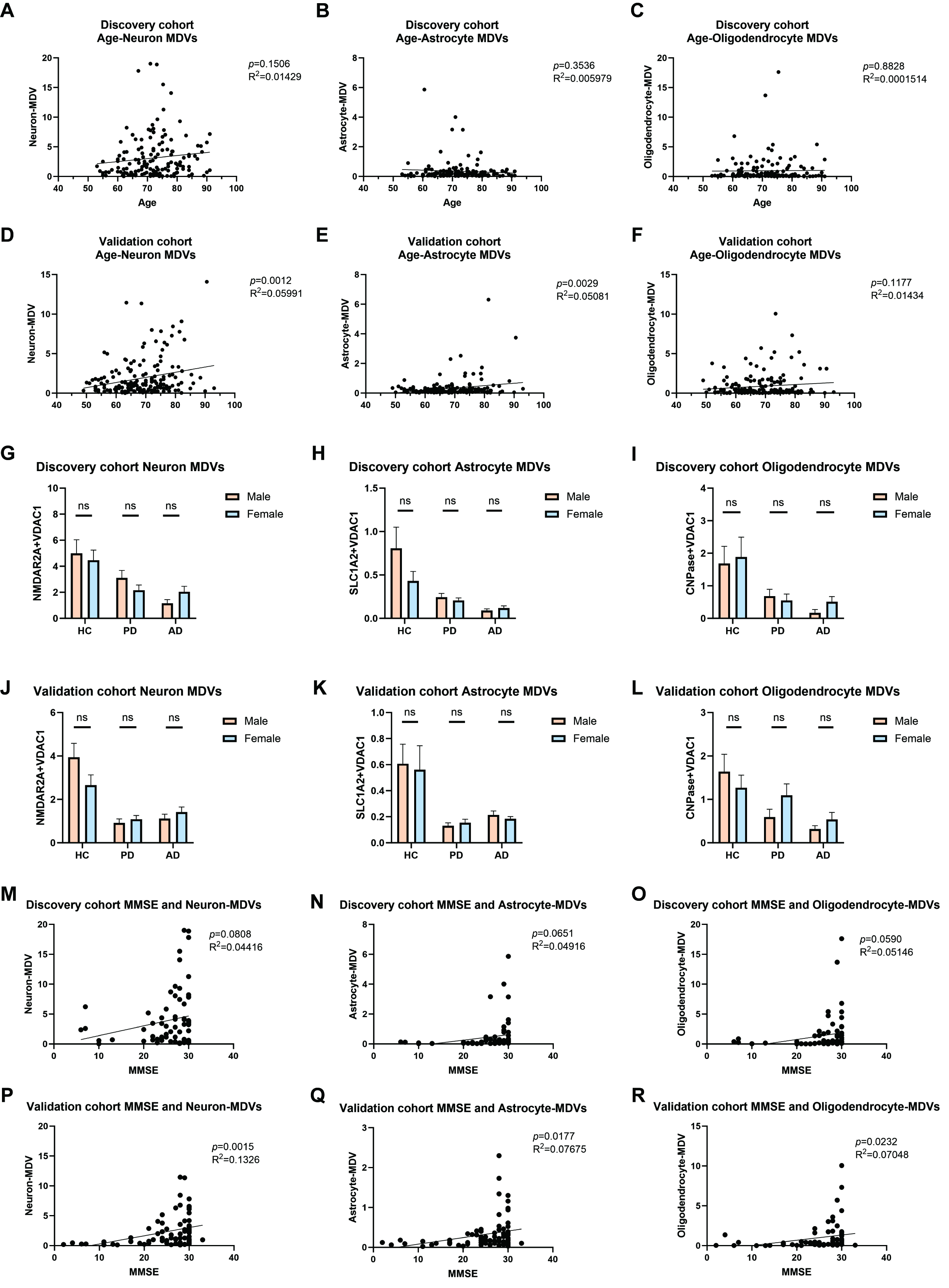

Supplement: Supplementary file 4 — Figure S4. Correlation between the level of CNS‐derived MDVs and age, gender, and MMSE. (A) Correlation between age and neuron‐derived MDVs in the discovery cohort. (B) Correlation between age and astrocyte‐derived MDVs in the discovery cohort. (C) Correlation between age and oligodendrocyte‐derived MDVs in the discovery cohort. (D) Correlation between age and neuron‐derived MDVs in the validation cohort. (E) Correlation between age and astrocyte‐derived MDVs in the validation cohort. (F) Correlation between age and oligodendrocyte‐derived MDVs in the validation cohort. (G) Neuron‐derived MDVs in female and male subjects of AD, PD patients, and HC in the discovery cohort. (H) Astrocyte‐derived MDVs in female and male subjects of AD, PD patients, and HC in the discovery cohort. (I) Oligodendrocyte‐derived MDVs in female and male subjects of AD, PD patients, and HC in the discovery cohort. (J) Neuron‐derived MDVs in female and male subjects of AD, PD patients, and HC in the validation cohort. (K) Astrocyte‐derived MDVs in female and male subjects of AD, PD patients, and HC in the validation cohort. (L) Oligodendrocyte‐derived MDVs in female and male subjects of AD, PD patients, and HC in the validation cohort. (M) Correlation between MMSE and neuron‐derived MDVs in the discovery cohort. (N) Correlation between MMSE and astrocyte‐derived MDVs in the discovery cohort. (O) Correlation between MMSE and oligodendrocyte‐derived MDVs in the discovery cohort. (P) Correlation between MMSE and neuron‐derived MDVs in the validation cohort. (Q) Correlation between MMSE and astrocyte‐derived MDVs in the validation cohort. (R) Correlation between MMSE and oligodendrocyte‐derived MDVs in the validation cohort. [file ACN3-12-1312-s008.tif]
